# Reduced Carbon Monoxide Saturation Coverage on Vicinal Palladium Surfaces: the Importance of the Adsorption Site

**DOI:** 10.1021/acs.jpclett.1c02639

**Published:** 2021-09-24

**Authors:** Fernando Garcia-Martinez, Elisabeth Dietze, Frederik Schiller, Dorotea Gajdek, Lindsay R. Merte, Sabrina M. Gericke, Johan Zetterberg, Stefano Albertin, Edvin Lundgren, Henrik Grönbeck, J. Enrique Ortega

**Affiliations:** † Centro de Física de Materiales CSIC/UPV-EHU-Materials Physics Center, Manuel Lardizabal 5, 20018 San Sebastian, Spain; ‡ Department of Physics and Competence Centre for Catalysis, Chalmers University of Technology, 41296 Göteborg, Sweden; § Department of Materials Science and Applied Mathematics, Malmö University, 21118 Malmö, Sweden; ∥ Synchrotron Radiation Research, Lund University, 22100 Lund, Sweden; ⊥ Combustion Physics, Lund University, Box 118, 22100 Lund, Sweden; # Departamento Física Aplicada, Universidad del País Vasco, 20018 San Sebastian, Spain; ∇ Donostia International Physics Centre, 20018 San Sebastian, Spain

## Abstract

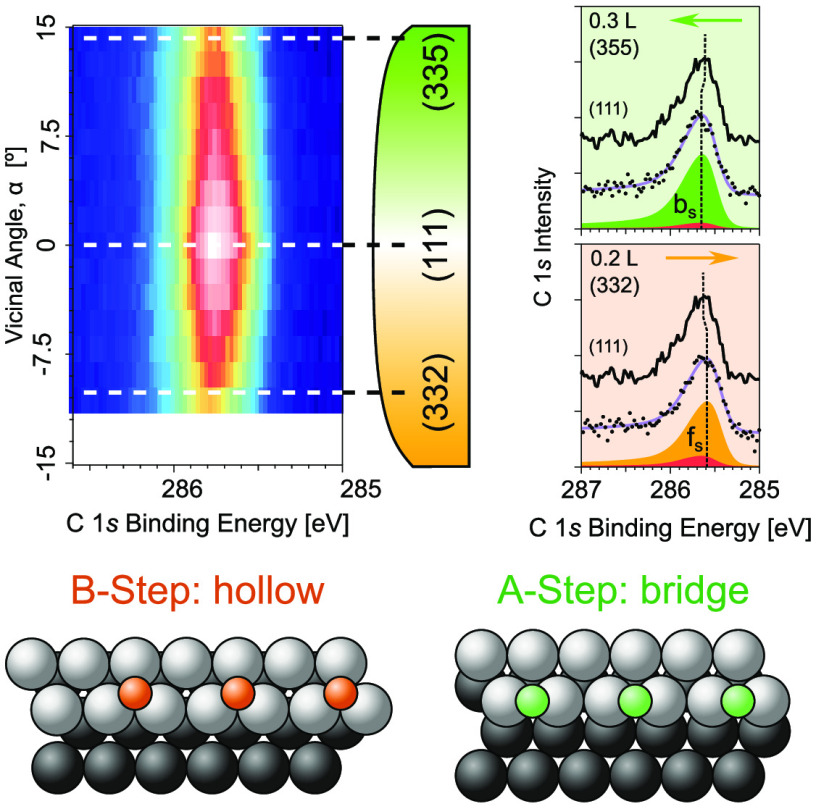

Steps at metal surfaces may influence energetics and kinetics of catalytic reactions in unexpected ways. Here, we report a significant reduction of the CO saturation coverage in Pd vicinal surfaces, which in turn is relevant for the light-off of the CO oxidation reaction. The study is based on a systematic investigation of CO adsorption on vicinal Pd(111) surfaces making use of a curved Pd crystal. A combined X-ray Photoelectron Spectroscopy and DFT analysis allows us to demonstrate that an entire row of atomic sites under Pd steps remains free of CO upon saturation at 300 K, leading to a step-density-dependent reduction of CO coverage that correlates with the observed decrease of the light-off temperature during CO oxidation in vicinal Pd surfaces.

Carbon monoxide (CO) adsorption on transition-metal surfaces is one of the elementary steps in the water–gas shift reaction, the Fischer–Tropsch synthesis, and the CO oxidation.^1−3^ CO oxidation is the canonical example of a Langmuir–Himshelwood process, in which the reactants, CO and O, interact at neighboring surface sites and eventually form CO_2_.^2,3^ In the presence of CO and O_2_ in the gas phase, coadsorption of CO and O is prevented at low temperatures, since CO saturates the surface and blocks the dissociative adsorption of O_2_. The CO saturating layer does not leave enough free surface space for O_2_ to impinge and react with coadsorbed CO. Thus, the temperature must be increased to facilitate CO desorption and creation of free surface sites for the reaction to start.^4^ Low temperature activity can be achieved by use of catalysts where the CO desorption energy is lowered, such as some bimetallic alloys,^5^ or bifunctional surfaces, such as oxide-supported nanoparticles, where CO and O_2_ adsorption occurs at physically separated nanoparticle and oxide sites.^6,7^

A vicinal metal surface may be viewed as a simple nanostructured catalyst, with different atomic coordination at terraces and steps, as well as a strong asymmetry in bonding configuration and lattice relaxation around the step edge.^8^ In the CO oxidation reaction, such a nanoscale structure of the vicinal surface is relevant since it affects the CO adsorption energy, which is higher at low coordination sites of steps as compared to the highly coordinated terraces.^9^ Therefore, close to the CO desorption temperature, one may expect CO-depleted terraces, where molecular oxygen adsorbs and reacts with CO adsorbed at steps.^10^ However, at a high step density, the simple terrace/step picture breaks down, because lower-coordination steps and higher-coordination corner-row atoms effectively involve a larger portion of the surface. The question that arises is how does this peculiar structural asymmetry of vicinal surfaces influence CO chemisorption in densely stepped planes, such as to affect the activation of the catalytic CO oxidation.

Here, we investigate the impact of atomic steps on the adsorption of CO on Pd(111), by monitoring the sequence of adsorption sites during CO uptake at vicinal surfaces at 300 K. To gain such information, we perform a full exploration of a curved Pd crystal sample using X-ray Photoelectron Spectroscopy (XPS) and Density Functional Theory (DFT). The Pd curved sample contains the (111) direction in the center of the surface and A- and B-type vicinal planes of increasing vicinal angle α at each side. Such a sequence of vicinal surfaces is probed with XPS (C 1s) after CO saturation at 300 K, showing the surprising behavior displayed in Figure 1a. The CO saturation coverage in Pd steadily decreases from the (111) center (α = 0) to the densely stepped sample edges by up to 30–40%, in contrast with the smaller 8–10% decrease found for the analogous curved Pt(111) surface. Our DFT-assisted analysis of the XPS spectra explains the Pd case: the lower (7-fold) coordination of step atoms leads to preferential CO adsorption in the upper part of the step [f_s_ in Figure 1a], followed by filling of terrace sites (9-fold coordination), up to the Pd corner-row in the lower part of the next step [11-fold for Pd(553)], which remains empty. This results in a linear decrease of the CO saturation coverage as a function of the step density, which correlates with the observed reduction in light-off temperature across Pd vicinal surfaces.^11,12^

**Figure 1:**
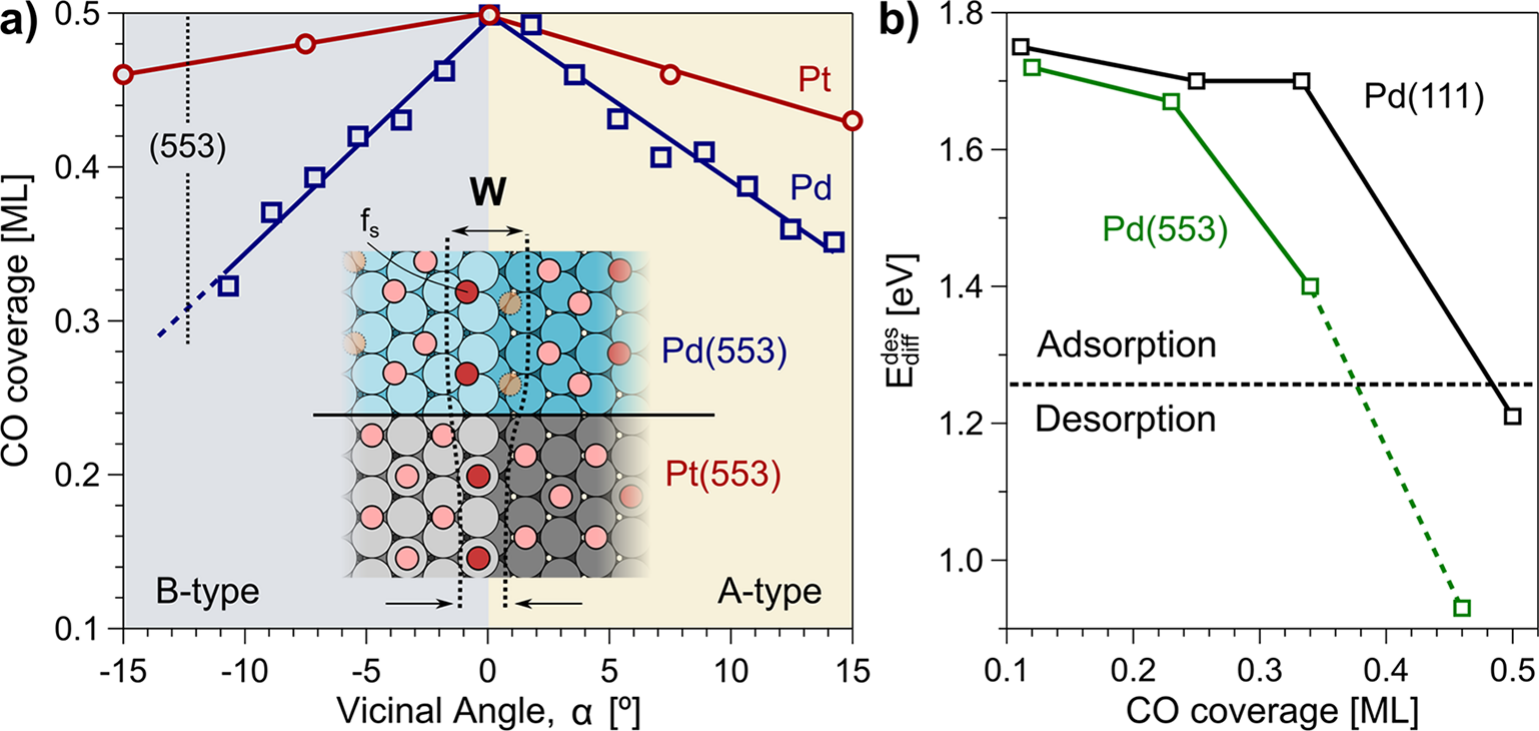
(a) CO saturation coverage at 300 K on Pd (blue) and Pt (red, ref (13).) vicinal surfaces, defined by the vicinal angle α with respect to (111). Lines are fit to data. The inset depicts the characteristic CO adsorption sites around monatomic B steps and marks the effective step width (*W*) during CO saturation for both Pt (smaller *W*) and Pd (larger *W*). (b) DFT-calculated differential desorption energy of CO, *E*_diff_^des^, as a function of coverage for Pd(553) and Pd(111). The horizontal dotted line marks the energy at which the desorption and adsorption rates are equal at 300 K and 10^–9^ mbar pressure.

Our curved sample is a cylindrical section (Δα = 30°) of a Pd single crystal, referred to as c-Pd(111), described in detail in ref (12) and in the Supporting Information (SI, Figure S1). It features the (111) surface at the center, the (553) plane (B-type {111} microfacet, α = −12.3°) close to one edge of the sample, and the (335) surface (A-type {100} microfacet, α = +14.4°) at the other edge. Ar^+^ sputtering, O_2_ annealing, and high-temperature flashes were employed for cleaning the sample. The cleanliness and surface structure of the sample were checked by Low Energy Electron Diffraction (LEED) and XPS prior to the CO dosing experiments. The LEED pattern reveals a smoothly variable density of monatomic steps away from the (111) direction.^11^

XPS experiments were carried out at the FlexPES beamlime of MaxIV synchrotron in Lund, Sweden. A photon energy of 400 eV was used to acquire C 1s spectra along the (111) normal, with the sample temperature kept at 300 K. Doniac-Šunjić lines^14^ convoluted with a Gaussian profile and a Shirley-type^15^ background were employed in the fitting procedure. As reported in the literature,^12,16,17^ two vibrational excitations of the adsorbed CO molecules were considered for each of the CO species included in the fitting routine (see section S2 of the SI for the peak deconvolution protocol). To support the core-level analysis, Density Function Theory (DFT) calculations were performed with the Vienna Ab-initio Simulation Package (VASP) implementation,^18−20^ using the PBE^21^ exchange-correlation functional and the projector-augmented wave (PAW) method^22,23^ to describe the interaction between the valence electrons and the core. The calculations use Pd(221) and Pd(112) as models for B- and A-types of steps, respectively. In addition, we use Pd(553) to study a surface with a large terrace and Pd(111) as a reference. Computational details and a full account of theoretical results are given in section S3 of the SI text, Tables S1–S5, and Figures S3–S5.

Room-temperature saturation is achieved after exposing the c-Pd(111) surface to a 10 Langmuir (L) CO dose at 300 K. The sample is thereafter vertically moved in front of the X-ray beam in discrete steps, corresponding to Δα = 1.75°, and the C 1s spectrum is acquired. The photoelectron intensity (area under the peak) is converted into coverage assuming the saturation of the (111) plane with 0.5 monolayers (ML), which is in turn cross-checked through its characteristic LEED pattern.^24^ The CO coverage variation across the c-Pd(111) saturated surface is shown in Figure 1a, compared to the corresponding variation on its analogous c-Pt(111) crystal.^13,25^ Lines are fit to data points and demonstrate that the total CO coverage decreases linearly as a function of the vicinal angle α, that is, the step density. Away from the (111) plane, the CO saturation coverage slightly decreases for platinum, while it strikingly drops in palladium, up to 30–40%, near the (335)–(332) vicinal planes of the A–B sides of the sample, respectively.

At fixed temperature, the CO saturation coverage is directly determined by the differential desorption energy, which depends on the CO–Pd bond strength and the (mainly) surface-mediated CO–CO repulsive interaction strength. Assuming a nonactivated desorption process (no desorption barrier), the desorption energy is taken to be equal to the converse of the adsorption energy. Across the stepped surfaces, the CO desorption energy is higher above the steps and decreases across the terrace toward the next step (see Table S3 in SI). The repulsive CO–CO interaction lowers the desorption energy dramatically in the dense layers, as reflected in Figure 1b. Here, we explore the coverage dependent desorption energy (*E*_diff_^des^) for the Pd(553) plane (see S3 section in SI for more details). The coverage in the calculations is stepwise increased by adding one CO molecule per 2 × 1 row along the (110) direction, starting from CO adsorbed at hollow fcc sites in the step edge (f_s_, 0.11 ML), followed by alternating bridge (b_t_) and fcc (f_t_) rows inside terraces, up to a fourth CO b_cr_ in the corner-row (0.46 ML). Filling the (553) surface with the corner-row makes the differential desorption energy fall below 1.0 eV (black dashed line), at which the CO adsorption rate is equal to the CO desorption rate at *T* = 300 K and 10^–9^ mbar pressure. Therefore, the corner row is empty in Pd(553), and the 300 K saturation coverage corresponds to three CO rows per terrace (nominally 0.34 ML). Note that the coordination number of corner-row surface atoms is 11, which implies that the CO desorption energy is reduced with respect to the (111) surface also in the absence of CO–CO repulsion. In contrast to the Pd(553) case, the desorption energy at Pd(111) is above the line for adsorption/desorption equilibrium close to 0.5 ML CO, demonstrating the difference in saturation coverage. On the other hand, atop and bridge adsorption configurations on the analogous Pt(553) stepped surface [see Figure 1a] allow higher saturation coverage without involving the highly coordinated corner-row atoms. This is a consequence of the spatial extension of the atop adsorption (one surface atom) as compared to the fcc adsorption (three surface atoms). We may define the effective step width *W* sketched in Figure 1a, as the relative surface area affected by CO adsorption at step sites in a vicinal surface, which is larger in Pd than in Pt. As shown below, experiments on the curved surface allows us to estimate *W* quantitatively.

A more detailed look into the C 1s spectrum during CO uptake at 300 K allows us to asses the sequence of CO bonding sites at different planes, and then postulate CO saturation geometries. For uptake experiments, the clean sample was exposed to 1 × 10^–9^ mbar CO while continuously monitoring the C 1s region. It is essential to first investigate the CO adsorption on the (111) plane, whose rich family of structural arrangements as a function of coverage and temperature has been a matter of discussion.^16,17,24,26−31^ Of particular relevance is the 0.5 ML saturation at 300 K,^16,24^ which corresponds to the coverage at which O_2_ can not adsorb even at near-ambient pressures.^12^ As shown in Figure 2a, at the lowest CO dose a single peak is detected at 285.62 eV [2b)], which we assign to CO adsorbed at hollow fcc sites at terraces (f_t_).^16^ f_t_ steadily grows with CO exposure up to 1.2 L, accompanied by a smooth shift to larger binding energy. At this point, and following Surnev et al.,^16^ the changing shape of the peak and its shift reveals the emergence of the b_t_ contribution (adsorption in bridge sites at terraces) at 285.77 eV.

**Figure 2:**
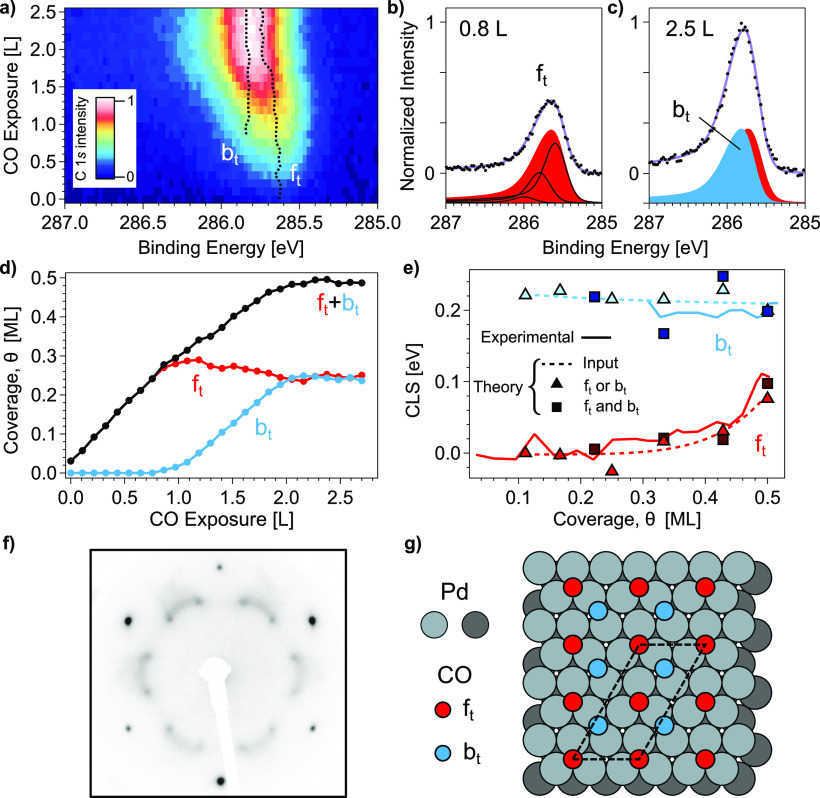
(a) C 1s intensity during CO uptake at the Pd(111) surface, acquired under 1 × 10^–9^ mbar CO dosage at 300 K. Photon energy is 400 eV. (b, c) Selected C 1s spectra at low (0.8 L CO) and high (2.5 L) CO exposures, together with fitting lines for f_t_ (red) and b_t_ (blue) components. Solid lines correspond to main and double satellite contributions, assumed for all peaks. (d) f_t_, b_t_, and total-CO (f_t_+b_t_) coverage evolution during the uptake shown in a. (e) Theoretical (squares, triangles) and experimental (solid lines) CLSs for f_t_ and b_t_ relative to the initial position of f_t_ in the (111) surface. Dotted lines are smooth fits to theory data. (f) LEED pattern from 10 L CO at Pd(111) (electron energy 80 eV). (g) Model proposed for 0.5 ML CO saturation of Pd(111) at 300 K.

Note that f_t_ and b_t_ peaks in Figure 2c are close and difficult to resolve. On the other hand, when the CO coverage approaches the 0.5 ML saturation, the mutual CO–CO repulsion increases (see Table S3 in SI). Thus, shifts in core-level binding energies are expected as they reflect the state of bonding.^32^ To obtain a reliable uptake curve [Figure 2d], we need to assist the line-fit analysis through the Core-Level Shift (CLS) DFT calculations shown in Figure 2e (symbols, see section S3 in SI for details). The calculations reveal that f_t_ and b_t_ do not shift equally as a function of coverage. Using an averaged theoretical curve (dotted line) as initial input to fit the experimental core levels, we obtain the solid lines, which represent experimental CLSs relative to the initial position of f_t_. These lines also appear overlaid in the color plots of Figure 2a. f_t_ shifts slightly by approximately +0.1 eV from 0.34 ML (corresponding to the (3^1/2^× 3^1/2^)R30° structure^16^) to saturation, while b_t_ remains virtually unchanged. The respective peak intensities, converted to CO coverage by normalizing to 0.5 ML at saturation, are represented in Figure 2d. Notably, this DFT-assisted fitting procedure leads to equal 50% contribution of b_t_ and f_t_ sites, in contrast to a larger b_t_ weight if the binding energy shift correction is not applied.

The structure of the CO-saturated (111) surface was probed with LEED. The 0.5 ML pattern shown in Figure 2f agrees with that of Ertl and Koch,^24^ although it appears better defined in our case. It has been argued that this pattern progressively “splits” from the (3^1/2^× 3^1/2^)R30° structure, due to the increasing presence of disordered domains from 1/3 to 0.5 ML.^16,32^ However, assuming the equivalent 0.25:0.25 ML b_t_/f_t_ coverage, we postulate the more simple *c*(4 × 2)-4CO arrangement sketched in Figure 2g. As discussed in section S2 in the SI, this structure directly produces most of the extinctions needed to generate the main spots of Figure 2g out of the fundamental *c*(4 × 2) pattern. Nevertheless, to explain the diffuse arc-shaped intensity without altering the b_t_/f_t_ ratio, there is a need to consider a certain degree of disorder, induced by the large CO mobility at 300 K, and also the presence of phase and antiphase *c*(4 × 2)-4CO domains (see section S2 and Figure S2 in SI).

The combination of XPS and DFT is powerful to extract detailed information from CO uptake curves at stepped surfaces. As an example, in Figure 3 we investigate the CO uptake at the B-type (332) and the A-type (335) surfaces of the c-Pd(111) sample. Color plots in Figure 3a,b correspond to C 1s spectra acquired in separate uptake experiments on both surfaces. Immediately after leaking CO into the chamber, a peak emerges at 285.66 and 285.60 eV in the (332) and (335) planes, respectively. As in other vicinal metal surfaces,^33−36^ we ascribe such an emerging line to CO adsorbed at steps. Although very slightly, peak energies are shifted toward lower (negative shift) and higher (positive shift) binding energy with respect to the f_t_ peak in the (111) plane at similar CO exposures, as shown in the left side panels of Figure 3c,d, respectively. The opposite shifts reflect a different adsorption site at the step edge, as revealed by the DFT calculation sketched in the respective right side panels. Here, we consider separately different CO adsorption sites at Pd(221) and Pd(112) surfaces, which are used to represent B- and A-type vicinal surfaces, respectively. All terrace sites exhibit positive shifts with respect to f_t_ on Pd(111), including step-bridge sites (b_s_). The hollow fcc site of the step (f_s_) has no shift on the A-type step, whereas the shift is negative on the B-type step. We therefore conclude that CO adsorbs at f_s_ step sites on the B-type Pd(332) surface and, as previously observed,^37,38^ at b_s_ sites on the A-type Pd(335).

**Figure 3:**
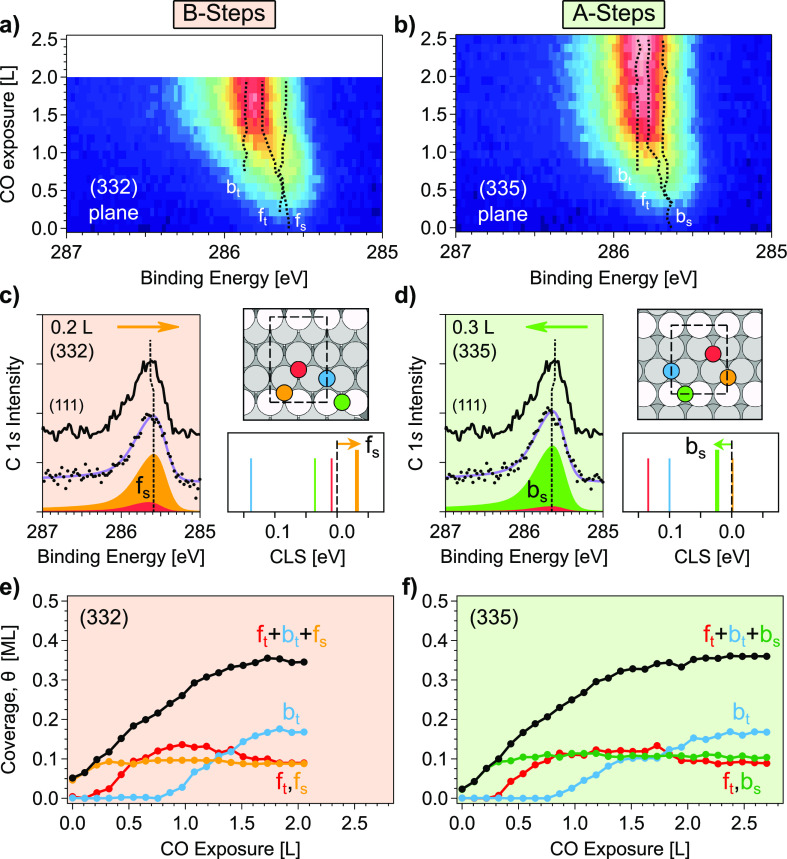
(a,b) C 1*s* intensity during CO uptake at the (332) and (335) surfaces, acquired under 1 × 10^–9^ mbar CO at 300 K. Photon energy is 400 eV and color scale is the same as in Figure 2. (c,d) Low CO dose spectra, with line fitting, for each of the aforementioned surfaces, compared to the same dose in Pd(111). The right side in each case shows the C 1s core-level shift (CLS) calculation, with respect to a single f_t_ molecule on the (111) surface, for CO on Pd(221) and Pd(112), respectively, in the sketched configurations. (e,f) CO coverage evolution during the CO uptake at the (332) and (335) surfaces, respectively.

At a higher CO dose, terrace f_t_ and b_t_ peaks emerge, leading to major changes in the C 1s features of Figure 3a,b. A minimal three-peak (f_t_, b_t_, and step) fit is thus required, which can reliably be done thanks to the curved surface systematics. The resulting binding energies for the three peaks are marked with dotted lines in Figure 3a,b, while the intensity (coverage) variation is shown in Figure 3e,f. Terrace and step peaks on both (332) and (335) surfaces evolve in a similar way. On the B-type (332) surface, the f_s_ step species saturates (0.5 CO/step atom, 0.094 ML) after exposure to 0.4 L CO. Above this dose, a second feature ascribed to f_t_ emerges. As observed on the (111) surface, b_t_ starts to grow when f_t_ reaches its maximum at 1.0 L, and f_t_ shifts toward larger binding energy as the system approaches saturation. At this point, both f_t_ and f_s_ have similar intensities, which is about half of the b_t_ intensity. On the A-type (335) surface, the b_s_ step peak saturates (0.5 CO/step atom, 0.088 ML) at 0.3 L, where the f_t_ emerges and grows up to its maximum at 0.8 L. From here, b_t_ grows and f_t_ slightly attenuates. On both (332) and (335) surfaces, the evolution of terrace f_t_ and b_t_ species is very similar to that observed in the (111) surface, although the relative intensity of b_t_ at saturation is significantly larger. This latter reflects the absence of CO at f_cr_ sites in the corner row, as discussed below.

Having identified the variety of the CO adsorption sites at the characteristic (332) and (335) vicinal planes, next we investigate the α-dependent evolution of each species in the CO-saturated c-Pd(111) surface. Figure 4a shows such an α-scan as a color plot. After exposing the clean sample to 10 L of CO at 300 K, individual C 1s spectra were systematically acquired at 15 different points on the curved surface. The plot nicely reflects the marked decrease of the CO layer coverage from the (111) center and toward the sample edges, represented in Figure 1a. All individual spectra are consistently fitted, following the analysis discussed in the previous section. As an example, we present spectra and fitting lines at three different sample points in Figure 4b. The resulting intensity variation of each component is shown in Figure 4c, together with the total “terrace-CO” (f_t_ + b_t_) signal (black markers). At α = 0, i.e., the (111) plane, f_t_ and b_t_ equally occupy the surface. Both decrease almost linearly as a function of α, as expected from the effective reduction of the occupied terrace area, due to both step and unoccupied corner-row Pd atoms. It is worth noting that b_t_ decreases at a lower rate as compared to f_t_, leading to the significant f_t_/b_t_ unbalance at the stepped edges, discussed in Figure 3. In contrast to terrace-CO, the intensity from f_s_ and b_s_ step species increases linearly away from the (111) center, and approximately at the same rate at A and B steps. This consistent step-peak intensity growth demonstrates that the number of CO molecules per step atom is not affected by the step density or type in this case.

**Figure 4:**
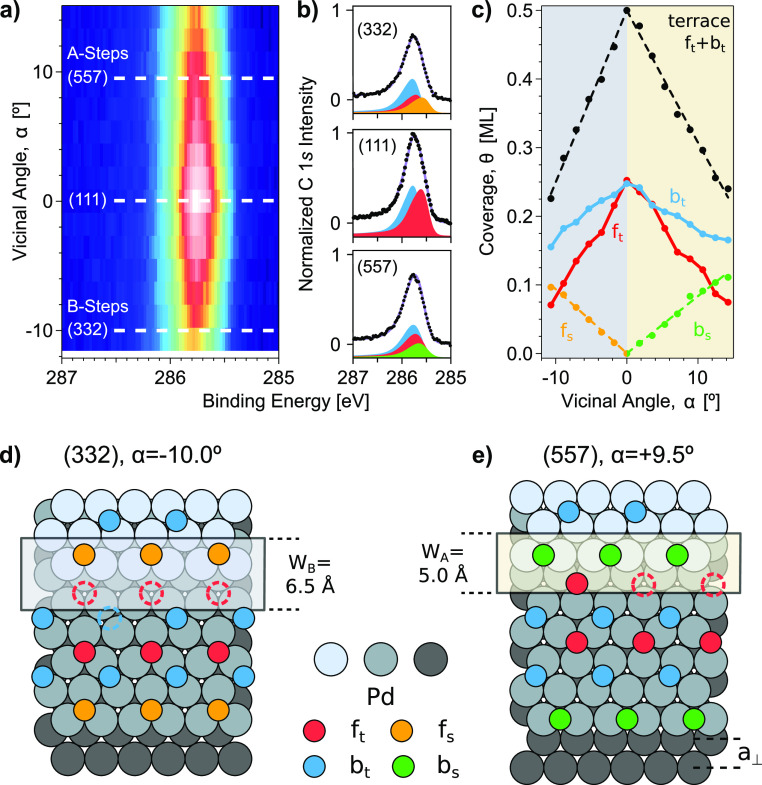
(a) C 1s α-scan across the CO-saturated c-Pd(111) surface, acquired after exposing the clean sample to 10 L of CO at 300 K. Photon energy and color scale as in Figure 2. (b) Individual spectra for relevant surfaces with the respective line-fitted components: terrace fcc-hollow (f_t_, red) and bridge (b_t_, blue) and step fcc-hollow (f_s_, orange) and bridge (b_s_, green). (c) Coverage of different CO species as a function of α. Black markers correspond to terrace-CO (f_t_+b_t_). The black dashed line fits terrace-CO data to the *W* model described in ref (39) and in section S1 of the SI. (d,e) CO layer saturation models proposed for the (332) and (557) surfaces. Dotted circles denote missing CO. The color code is the same as in b and c.

To asses the effective width of the step *W*, that is, the surface area affected by CO adsorption at steps [see Figure 1a], we fit the total terrace-CO and step-CO intensities using the *W*-model described in ref (39) and section S1 of the SI. Assuming monatomic steps (2.25 Å height) and a fixed terrace-CO density of 0.5 ML, the model renders the effective step width *W*_A_ = 6.5 Å and *W*_B_ = 5.0 Å, for A and B steps. It also yields Θ_S,A_ = 0.39 and Θ_S,B_ = 0.44 molecules per step atom, close to the expected 0.5 ratio. *W*_A_ and *W*_B_ can be compared to the distance between Pd atomic rows (*a*_⊥_, 2.75 Å), revealing that the effective width of A- and B-type steps exceeds, by 136% and 82%, that of the atom row *a*_⊥_, respectively. Therefore, CO occupation of step sites not only affects the step-atom row but also blocks CO adsorption within a significant portion of the terrace, namely the corner row. As discussed above, CO adsorption at bridge or hollow positions in the corner-row leads to a large increase in the intermolecular repulsion, and hence corner-row Pd atoms remain CO free.

In Figure 4d,e, we postulate the CO layer structure at saturation for characteristic B-type and A-type vicinal Pd planes, namely, Pd(332) and Pd(557), respectively. In essence, we first complete the 0.5 molecule-per-atom step edge and then fill up alternating b_t_ (blue) and f_t_ (red) rows as we move into the terrace. Following Figure 3e,f results, the filling process begins with f_s_ (orange) or b_s_ (green) sites at the (332) or (557) plane, respectively, and extends to the terrace until the total (experimental) saturation coverage is reached in each case, 0.34 ML for Pd(332) and 0.40 ML for Pd(557). On the basis of the theoretical analysis of Figure 1b, the entire row of fcc hollow sites in the corner-row remains empty in the (332) surface, while in the (557) surface three out of four such corner-row sites are unoccupied. Interestingly, such a small difference in corner-row occupation at A- and B-type steps could also be expected from their distinct atomic coordination and the small adsorption energy difference that it implies: on A-type surfaces, such as Pd(557), the hollow site at the corner row involves CO bonding to a 10-fold coordinated Pd atom. The resulting CO desorption energy is therefore slightly higher than the one expected at the B-type Pd(332) surface, where corner-row Pd atoms are 11-fold coordinated.

In summary, vicinal surfaces possess asymmetric bonding configurations around steps, with metal atoms having lower and higher coordination at step and corner atoms, respectively, as compared to terrace atoms. Here, we have investigated how this property alters CO adsorption, particularly at densely stepped surfaces. Our XPS intensity profile of a curved Pd(111) surface saturated with CO at 300 K demonstrates that Pd vicinal surfaces exhibit a significant reduction of the saturation coverage as a function of the step density, which DFT calculations link with the effective elimination of the CO adsorbed at corner-row atoms. This nonuniform coverage scenario is confirmed by the analysis of the C 1s core level during CO uptake at 300 K, performed at different vicinal planes of the curved Pd substrate. With the assistance of DFT calculations, which facilitate the identification of the different core-level contributions, CO uptake curves reveal that the characteristic 1:1 bridge/hollow balance of the (111) surface is broken at terraces in vicinal surfaces, owing to empty unfavorable hollow sites at the corner row. A point-by-point analysis of the saturation spectra at the curved surface confirms the steady reduction of terrace hollow sites with respect to bridge sites as a function of the step density in both A- and B-type vicinal surfaces.

## Supporting Information Available

Description of the Pd curved sample used in the experiments; description of the *W* model to estimate the effective step width; line fit and peak deconvolution protocol; LEED simulations for several 0.5 ML structures of CO chemisorbed on Pd; CO adsorption on Pd; CO desorption; C 1s core-level shift; (Figure S1) sketch of the curved Pd sample; (Figure S2) 0.5 ML structures and calculated LEED patterns; (Table 1) CO/Pd surface models for DFT calculations; (Table 2) adsorption energies at different configurations; (Table 3) coverage dependent mean adsorption energies; (Table 4) C 1s core-level shift for different CO coverage on Pd(111); (Table 5) C 1s core-level shift at low CO coverage on Pd(112) and Pd(221); (Figure S3) adsorption rates for CO on Pd(111) at 300 K; (Figure S4) adsorption sites on the (111), (112), (221), (553), and (332) surfaces; (Figure S5) sketch for the C 1s core-level shift calculation; graph for the C 1s core-level shift versus adsorption energy (PDF)
