# Supplementary material for: Reduced Carbon Monoxide Saturation Coverage on Vicinal Palladium Surfaces: the Importance of the Adsorption Site
Source: J Phys Chem Lett. 2021 Sep 24;12(39):9508–15. doi: 10.1021/acs.jpclett.1c02639 (PMC8503880; doi:10.1021/acs.jpclett.1c02639)
Supplement: Supplementary file 1 — jz1c02639_si_001.pdf [file jz1c02639_si_001.pdf]

Reduced Carbon Monoxide Saturation Coverage on Vicinal Palladium Surfaces: the Importance of the Adsorption Site

Fernando Garcia-Martinez,[†] Elisabeth Dietze,[‡] Frederik Schiller,[†] Dorotea Gajdek,[¶] Lindsay R. Merte,[§,¶] Sabrina M. Gericke,[‖] Johan Zetterberg,[‖] Stefano Albertin,[§] Edvin Lundgren,[§] Henrik Grönbeck,[‡] and J. Enrique Ortega[*,†,⊥,#]

†*Centro de Física de Materiales CSIC/UPV-EHU-Materials Physics Center, Manuel Lardizabal 5, 20018 San Sebastian, Spain*

‡*Department of Physics and Competence Centre for Catalysis, Chalmers University of Technology, 41296 Göteborg, Sweden*

¶*Department of Materials Science and Applied Mathematics, Malmö University, 21118 Malmö, Sweden*

§*Synchrotron Radiation Research, Lund University, 22100 Lund, Sweden*

‖*Combustion Physics, Lund University, Box 118, 22100 Lund, Sweden*

⊥*Departamento Física Aplicada, Universidad del País Vasco, 20018 San Sebastian, Spain*

#*Donostia International Physics Centre, 20018 San Sebastian, Spain*

E-mail: enrique.ortega@ehu.es

Contents

S1 Supplementary Information 2

S2 Supplementary data and analysis 3

S3 Density functional theory calculations 5

References 17

S1 Supplementary Information

S1.1 Curved Pd sample

The curved Pd sample [c-Pd(111)], sketched in Figure S1, was described in Ref. 1. It is a cylindrical section of a Pd single crystal featuring the (111) plane at the center and the cylinder axis parallel to the $[1\bar{1}0]$ direction. This geometry leads to vicinal surfaces with increasing density of close-packed steps as one moves away from the sample center to the edges, that is, A-type vicinals ({100} microfacet steps) for positive α angles, and B-type vicinals ({111} microfacets) for negative α, up to $\Delta\alpha = \pm 16^{\circ}$. Ar^+ sputtering (1 keV, $5{\cdot}10^{-7}$ mbar, I_{em}= 15 mA), O_2 annealing ($1{\cdot}10^{-7}$ mbar, 200-700 ºC) and short annealing flashes (800 ºC, 5 min) were employed to clean the sample, until no contamination was detected by means of XPS, and a smoothly increasing splitting of the hexagonal LEED pattern away from the (111) plane was obtained.[2–4] The split-pattern remains stable upon CO dosing at 300 K, proving the high stability of the monatomic step array. Beam-damage leads to CO cracking and graphitic carbon build-up, as revealed by the characteristic C 1s satellite at 284 eV.[4] The intensity (area under the peak) of graphitic carbon, though, remains below 5% of the total C 1s emission at every point on the curved sample.

S1.2 W-model

The step-size-W model is used to evaluate, out of the experimental photoemission intensity variation across the curved surface, the effective area of the vicinal surface affected by adsorption at atomic steps (W). The model assumes constant CO layer structure and coverage at terraces and steps at every vicinal angle α,[4] leaving the lateral extension of the step W as a fitting parameter. On these grounds, it is easy to see that terrace (Θ_T) and step (Θ_S) coverages must vary as as a function of α as:

$$\Theta_T(\alpha) = \Theta_T^0 \left[1 - W \cdot d^{-1}\right] = \Theta_T^0 \left[1 - W \cdot \frac{\sin(|\alpha|)}{h}\right] \tag{1}$$

$$\Theta_S(\alpha) = \Theta_S^0 \cdot W \cdot d^{-1} = \Theta_S^0 \cdot W \cdot \frac{\sin(|\alpha|)}{h} \tag{2}$$

where Θ_T^0 and Θ_S^0 refer to the constant saturation coverage at (111) terraces (0.5 ML at 300 K[5]) and steps, while d^{-1} and h stand for the step density and the height of a Pd monoatomic step (2.25 Å). By consistently fitting α-dependent terrace and step peak intensities to Equation 1 and Equation 2, W and the step coverage Θ_S^0 are readily determined. Since $d^{-1} = \sin(|\alpha|)/h$, both equations are expressed in terms of the step density or the vicinal angle.

S2 Supplementary data and analysis

S2.1 0.5 ML CO on Pd(111) LEED simulation

LEED patterns for several structures were simulated using the kinematic approximation, to explore the qualitative effects of various CO site occupations and arrangements in comparison to that of a $c(4{\times}2)$ phase involving hollow ($\mathrm{f_t}$, $\mathrm{h_t}$) and bridge $\mathrm{b_t}$ sites. Standard in-plane kinematic structure factors were calculated for each phase using the form factor of a carbon atom and a shape factor corresponding to Gaussian beams of width 0.15 Å^{-1}. Each pattern

shown in Figure S2 is the average from the set of equivalent domains expected based on the substrate p3m1 symmetry. All of them correspond to 0.5 ML coverage with hollow/bridge=1 ratio, as deduced from the XPS fitting (Fig. 2 of the main text). For the ease of comparison, the original $c(4\times2)$-4CO spots are indicated with red circles, while the connecting arcs of the saturation superstructure (Fig. 1 in the main text) are marked with blue shades. The highest symmetry $c(4\times2)$-4CO superstructure is shown in Figure S2a), showing the expected diffraction spots. If antiphase domains are considered [Figure S2b)], a significant amount of spots are still observed at lower wavevector q than those of the original structure. Several low-q spots arise when tilted domain boundaries are considered [Figure S2c)], yet the spots split, resembling our arc-like pattern. If vertical domains are simulated [Figure S2d)], the low-q spots remain, although the main spots split, although in the opposite direction as ours. Finally, when a random site occupation is simulated [Figure S2e)], only the main spots of the $c(4\times2)$ structure arise, since most of the lower-q reflections no longer appear in the simulation. Therefore, we propose the formation of several disordered domains of $c(4\times2)$-4CO superstructures, which would give rise to the pattern that we observed at Pd(111).

S2.2 Deconvolution of core level spectra

The fitting procedure was performed using the *lmfit* Python package.[6] We considered Doniac-Šunjić lines[7] convoluted with a Gaussian profile and a Shirley-type[8] background for the model. As reported in the literature,[1,9,10] two vibrational excitations of the adsorbed CO molecules were considered for each of the CO species included in the fitting routine. Such satellites were fixed at +0.2 and +0.4 eV above the major contribution, while their height were fixed at 45% and 9% of that of the main line. There parameters were extracted from the fitting of the (111) surface after CO saturation. Assuming an equal behaviour for all peaks, very similar factors (*i.e.* asymmetry, width, satellites) were used for all CO species discussed in the manuscript. The area of the satellites was added to that of the major contribution in order to properly calculate the coverage of each of the individual CO adsorption sites.

The fitting routine for the stepped surfaces is not straightforward, since the peaks overlap and are difficult to distinguish. In order to reasonably extract each species, we added a single line (step-CO peak) at the beginning of the CO uptake curves (Fig. 3 of main text). Such contribution was allowed to grow until a step coverage of 0.5 ML was reached (*i.e.* one CO per two Pd step atoms). After saturation of steps, the parameters of the step peak were held constant (only small variations were allowed), and an additional line (f_t) was included in the fitting procedure. Mimicking the (111) plane uptake shown in Fig. 2 of the main text, a third peak (b_t) was added when the overall spectrum started to shift towards larger binding energy.

S3 Density functional theory calculations

Density function theory (DFT) calculations have been performed with the Vienna Ab-initio Simulation Package (VASP) implementation,[11–13] using the PBE[14] exchange-correlation functional and the projector-augmented wave (PAW) method[15,16] to describe the interaction between the valence electrons and the core. The Brillouin zone was sampled using the Monkhorst-Pack[17] scheme.

The lattice parameter for Pd (3.946 Å) was obtained by optimizing the bulk unit cell with a 800 eV kinetic energy cut-off and a (16,16,16) k-point sampling. A 450-eV kinetic energy cut-off was used for the slab calculations. The calculations were done with a Gaussian smearing using $\sigma = 0.1$. The considered surface models are summarized in Table 1 reporting the surface cell, total number of layers (N_{Layer}), number of frozen layers (N_{frozen}) and k-point sampling. Repeated slabs were separated by at least 12 Å vacuum. Structures were optimized until the forces were below 0.01 eV/Å.

S3.1 CO adsorption on Pd

It is experimentally established that the stable low-coverage site for Pd(111) is the fcc/hcp hollow site. In contrast to Pt(111),[18–20] the hierarchy of adsorption energies calculated with PBE for Pd(111) is in agreement with the experimental observation. The issue for Pt(111) has been traced to an underestimation of the HOMO-LUMO (5σ-and $2\pi^*$) separation in CO using conventional approximations to the exchange-correlation functional and the unbalanced 5σ- and $2\pi^*$-contributions to the CO-metal bond is present also for Pd(111). One sign that the bond is not properly described is that the bridge position is only a shallow minimum on the potential energy surface. Thus, all structures with CO in bridge position on the Pd surfaces have been obtained by constraining the C and O atoms to the center of the bridge positions. The CO adsorption energy is calculated as:

$$E_{\text{ads}} = E_{\text{CO,metal}} - E_{\text{CO}} - E_{\text{metal}} \tag{3}$$

with $E_{\text{CO,metal}}$ being the total energy of the adsorbed structure, E_{CO} the molecular gas-phase reference and E_{metal} the metal surface reference. Exothermic adsorption implies that the adsorption energy is negative. The corresponding desorption energy is the converse of the adsorption energy and, hence, positive for exothermic adsorption.

Table 2 shows the calculated adsorption energies for CO on the different surface sites together for Pd(111). The zero-point energy correction is not included in the adsorption energies but reported separately. Experimental values are taken from Ref. 21 where isosteric heats of adsorption were evaluated from the adsorption isotherms in the zero coverage limit.

The description of small gas-phase species is often an issue calculations using semi-local functionals, which causes problems when evaluating adsorption energies as the cancellation of errors is limited. One pragmatic way to reduce the problem is to shift the gas-phase energies so the heat of formations for a set of gas-phase reactions agree with tabulated experimental values.[22] We have adopted this approach and minimized the mean absolute

error and maximal error by correcting the gas-phase total energies for 10 gas-phase reactions including CO, H_2 and O_2.[22] The total energies for gas-phase CO, H_2 and O_2 are in this way shifted by -0.290 eV, +0.153 eV and +0.287 eV, respectively. Only the value for CO is used in this work. Because the calculated zero-point energy for CO adsorbed in bridge and fcc site on Pd(111) is similar, we use an overall shift of +0.346 eV to adsorption energies evaluated without zero-point correction and gas-phase correction (as reported in Table 3) to the values in Figure 1b in the main text.

CO adsorption has been explored for a range of different surfaces and coverages. The resulting adsorption and differential adsorption energies are listed in Table 3. For the stepped surfaces, different adsorption sites across terraces have been considered, as schematically depicted in Fig. S4.

S3.2 CO desorption

To determine the desorption energy for CO at the experimental conditions and compare it to the calculated values (Fig. 1b, main text), we calculated the rate constant for desorption from the adsorption rate constant and the equilibrium constant:

$$k_{\text{ads}} = \frac{p \cdot s_0 \cdot A_{\text{site}}}{\sqrt{2 \cdot \pi \cdot m \cdot k_{\text{B}} \cdot T}} \tag{4}$$

$$K = \exp(-\Delta H/(k_{\text{B}} \cdot T))\exp[(S_{\text{surf}} - S_{\text{gas}})/k_{\text{B}}] \tag{5}$$

$$k_{\text{des}} = \frac{k_{\text{ads}}}{K} \tag{6}$$

where p is the CO pressure, $s_0 = 0.9$ the sticking coefficient, A_{site} the surface site area, ΔH the corrected enthalpy of adsorption, S_{surf} the entropy of the adsorbate and S_{gas} the entropy of the molecule in that gas-phase. Figure S3 shows the adsorption/desorption rate versa desorption energy for different CO pressures. Lower CO pressures shift the desorption energy to higher values.

S3.3 C 1s core level shift

To identify possible adsorbate configurations on the stepped Pd surfaces, we calculated the C 1s core level shift (CLS) of CO adsorbed in different positions and at different coverages. To compare the CLS between stepped and flat surfaces, shifts were calculated with respect to a carbon atom placed deep in the bulk of the slab. To reduce the interaction between the carbon atom placed in the bulk and the slab surfaces, the slab thickness was increased to at least 8 layers. CLS's were calculated including screening of the core hole.[23] In this final-state approach, the CLS is calculated as a total energy difference between systems with a core-ionized atom replacing either the atom of interest or the reference atom:

$$\mathrm{CLS} = E^{\mathrm{surf}}_{\mathrm{C1s}} - E^{\mathrm{ref}}_{\mathrm{C1s}} \tag{7}$$

$E^{\mathrm{surf}}_{\mathrm{C1s}}$ is the total energy with the C 1s core-hole in one of the adsorbates on the surface and $E^{\mathrm{ref}}_{\mathrm{C1s}}$ is the total energy with the C 1s core-hole in the reference carbon atom. A schematic representation of the used structure is given in Figure S5a. Because the same super-cell structure is used to calculate $E^{\mathrm{ref}}_{\mathrm{C1s}}$ and $E^{\mathrm{surf}}_{\mathrm{C1s}}$ it is not necessary to calculate the ground state energy of the unperturbed structure. The calculations with core-ionized atoms were done using neutral super-cells, by adding an extra electron to the valence electrons.

The CLS is related to the adsorption energy in the Z+1 picture.[24] In Figure S5b we represent the CLS relative to the low-coverage fcc on Pd(111) against the adsorption energy. For CO adsorption on (111) transition metal surfaces, a linear trend of the CLS with coverage has been reported previously,[25–27] as well as for ethylene adsorption on Pd clusters.[28] The linear trend is also found for the stepped surfaces in the present work. The data for the coverage dependence of the CLS on Pd(111) is reported in Table 4. For the low coverage on Pd(112) and Pd(221), the data is reported in Table 5.

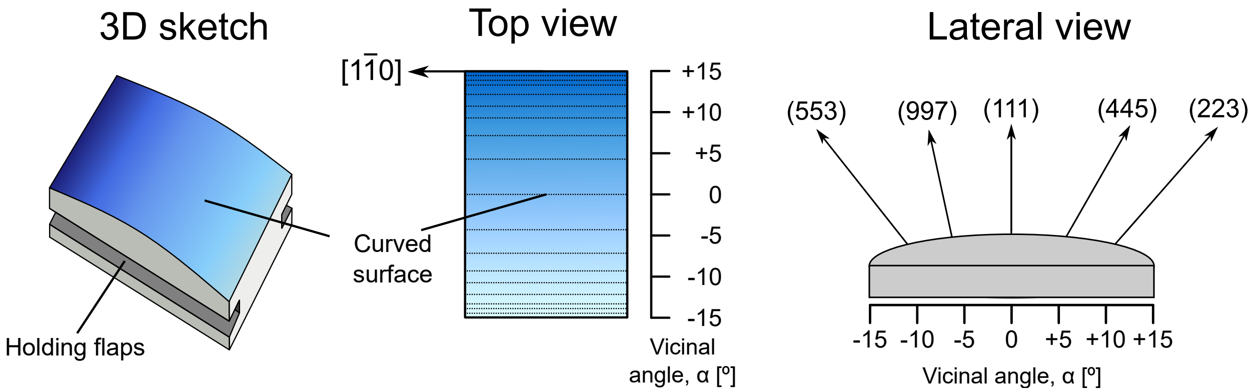

Figure S1: Different views of the c-Pd(111) sample with selected Miller indexes across the curved surface. The horizontal dashed lines in the top view illustrate the increasing step density as one approaches the edges of the crystal. The vicinal angle, α, is defined with respect to the (111) surface at the center. Assuming monoatomic steps, α is related to the step density (d^{-1}) as $d^{-1} = sin|\alpha|/h$.[1,2,4,29]

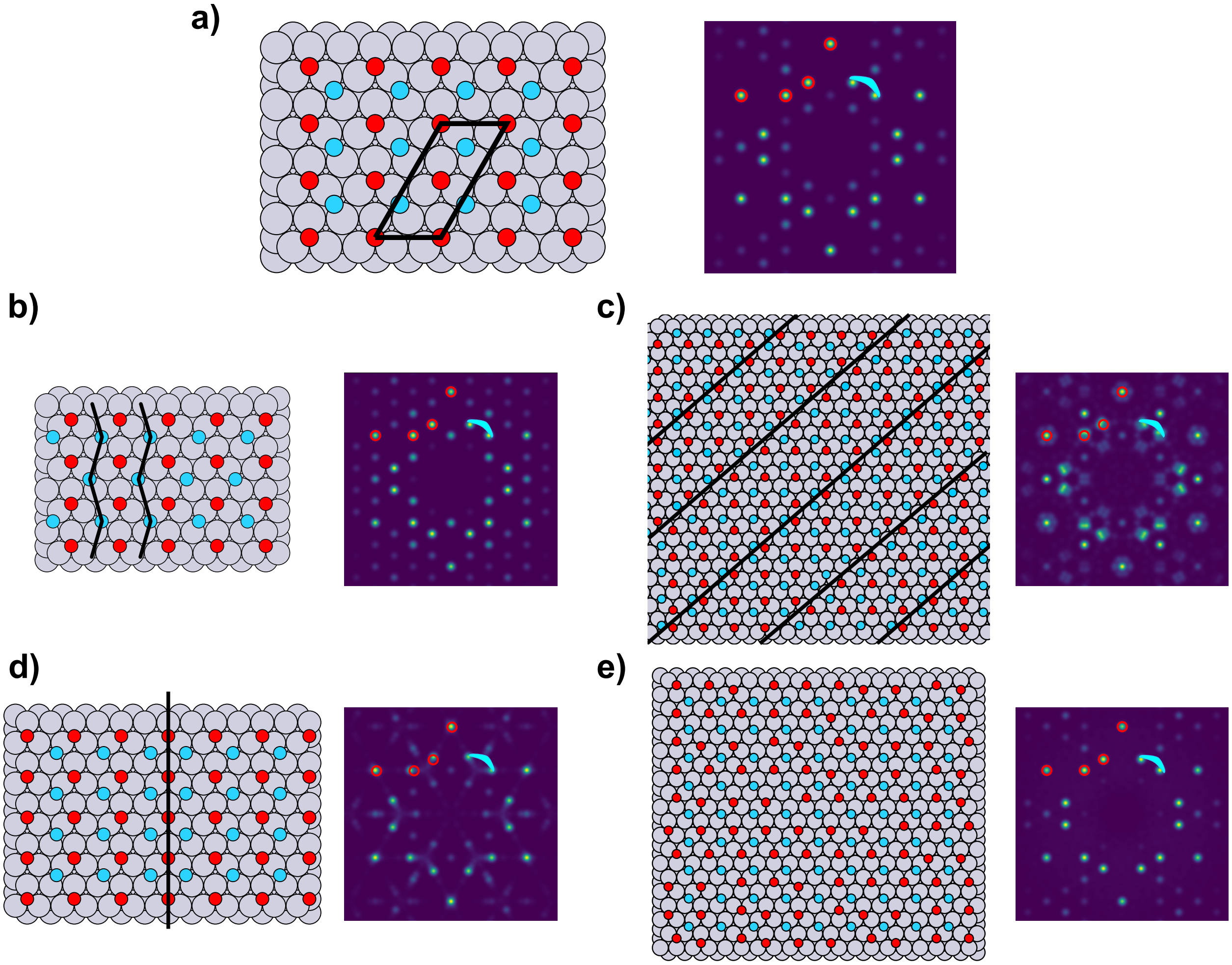

Figure S2: 0.5 ML, 1:1 f_t:b_t superstructures in real space (left) and their corresponding LEED pattern simulations (right). The structure sketched in **a)** corresponds to a $c(2 \times 2)$-2CO superstructure, while that of **b)** reflects the same superstructure with alternating anti-phase domains. **c)** combines tilted domains of the aforementioned structure, whilst **d)** corresponds to two vertical domains of the original superstructure. Finally, **e)** shows the simulation considering random occupation of sites (note the extinction of low-q spots).

Table 1: Considered surface models with applied surface cells, total number of layers (N_{Layer}), number of frozen layers (N_{frozen}) and k-point sampling.

Surface	surface cell	N_{Layer}	N_{frozen}	k-points
111	$(\sqrt{3} \times 2)$ Rect.	4	2	(6,6,1)
111	(3×3)	4	2	(6,6,1)
111	$(\sqrt{3} \times \sqrt{3})$	4	2	(6,6,1)
112	(2×1)	4	2	(6,6,1)
221	(2×1)	4	2	(6,4,1)
553	(2×1)	5	3	(6,3,1)

Table 2: Adsorption energies with zero-point corrections for CO on Pd(111) for the different adsorption sites as obtained with PBE. The calculations are done in a (3×3) surface cell with a coverage of $1/9$. The zero-point correction is reported explicitly. The adsorption energy including gas-phase corrections (including zero-point corrections) is reported in column 4. The experimental value corresponds to the low coverage limit.[21] The result for the bridge site is obtained by constraining the C and O atoms to the center of the bridge position.

Site	E_{ads} [eV]	ZPE [eV]	$E_{\text{ads}}^{\text{corr.}}$	Experiment [eV]
atop	-1.458	0.189	-1.115	
bridge	-1.901	0.190	-1.557	
fcc	-2.095	0.192	-1.749	-1.47
hcp	-2.074	0.214	-1.706	-1.47

Table 3: Coverage dependent mean adsorption energies E_{ads} and differential desorption energies $E_{\mathrm{diff}}^{\mathrm{des}}$ for Pd (111), (112), (221) and (553) surfaces. For the (111) surface, the employed super-cell is given in brackets: o. for the orthogonal, h. for hexagonal and s. for $\sqrt(3)x\sqrt{3}$-cell.

Surface	Coverage [ML]	Adsorption site	E_{ads} [eV]	$E_{\mathrm{diff}}^{\mathrm{des}}$ [eV]
111	0.111 (h.)	fcc	-2.095	2.095
	0.25 (o.)	fcc	-2.045	2.045
	0.333 (s.)	fcc	-2.041	2.041
	0.5 (o.)	fcc bridge	-1.803	1.560
112	0.167	b_e	-1.983	
	0.167	f_{mr-t}	-1.844	
	0.167	f_{e-t}	-1.973	
	0.167	h_{e-t}	-2.048	
	0.167	b_t	-1.868	
221	0.125	b_e	-1.942	
	0.125	f_{mr-t}	-1.782	
	0.125	f_t	-2.044	
	0.125	f_{e-t}	-2.085	
	0.125	h_t	-2.014	
	0.125	b_t	-1.888	
553	0.1	f_{e-t}	-2.071	2.071
	0.1	f_t	-2.028	
	0.1	h_{mr}	-1.523	
	0.1	h_{t2}	-2.027	
	0.2	f_{e-t} f_t	-2.043	2.014
	0.2	f_{e-t} b_t	-2.006	1.941
	0.3	f_{e-t} b_t f_t	-1.933	1.788
	0.4	f_{e-t} b_t f_t b_{t2}	-1.831	1.524

Table 4: C 1s CLS for different CO coverage on Pd(111). f and b stand for fcc and bridge, respectively. The shifts are computed with respect to CO at fcc with 0.111 coverage (f^0).

Position	Coverage	CLS(f)	CLS(b)	$\Delta(b - f^0)$	$\Delta(f - f^0)$
fcc	0.111	0.459			0
bridge	0.111		0.680	0.221	
fcc,bridge	0.222	0.464	0.678	0.219	0.006
fcc	0.167	0.456			-0.003
bridge	0.167		0.686	0.227	
fcc	0.250	0.433			-0.026
bridge	0.250		0.674	0.215	
fcc	0.333	0.475			0.016
bridge	0.333		0.674	0.215	
fcc,bridge	0.333	0.480	0.626	0.167	0.021
fcc	0.429	0.489			0.031
bridge	0.429		0.687	0.229	
fcc,bridge	0.429	0.478	0.706	0.248	0.019
fcc	0.500	0.535			0.076
bridge	0.500		0.658	0.199	
fcc,bridge	0.500	0.556	0.657	0.198	0.098

Table 5: Core level shifts of low coverage Pd(112) and Pd(221). The last columns gives the difference between the calculated CLS and the low coverage limit Pd(111) fcc C 1s.

Surface	Position	Coverage	CLS	$\Delta(\mathrm{CLS} - f^0_{111})$
221	fcc edge	0.125	0.427	-0.032
221	fcc terrace	0.125	0.467	0.009
221	fcc missing row	0.125	0.575	0.116
221	hcp terrace	0.125	0.511	0.052
221	bridge edge	0.125	0.494	0.035
221	bridge terrace	0.125	0.597	0.138
112	fcc edge	0.167	0.458	-0.001
112	fcc terrace	0.167	0.593	0.134
112	bridge edge	0.167	0.482	0.024
112	hcp edge	0.167	0.424	-0.035
112	bridge terrace	0.167	0.559	0.100

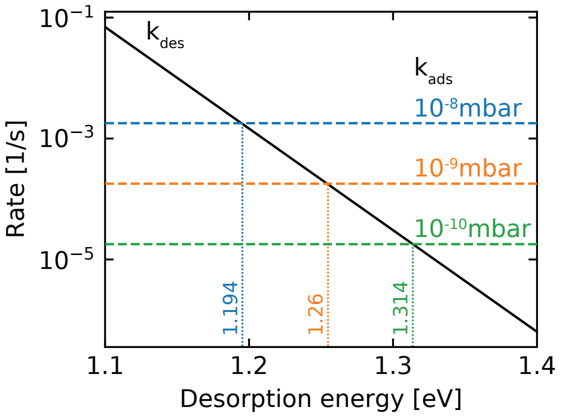

Figure S3: Adsorption rates for $p_{\mathrm{CO}} = 10^{-8}$, 10^{-9} and 10^{-10} mbar and $T = 300$ K calculated according to eq. (4) and desorption rate (eq. (6), black solid line). The corresponding desorption energies in eV at which $k_{\mathrm{ads}} = k_{\mathrm{des}}$ are indicated as vertical lines.

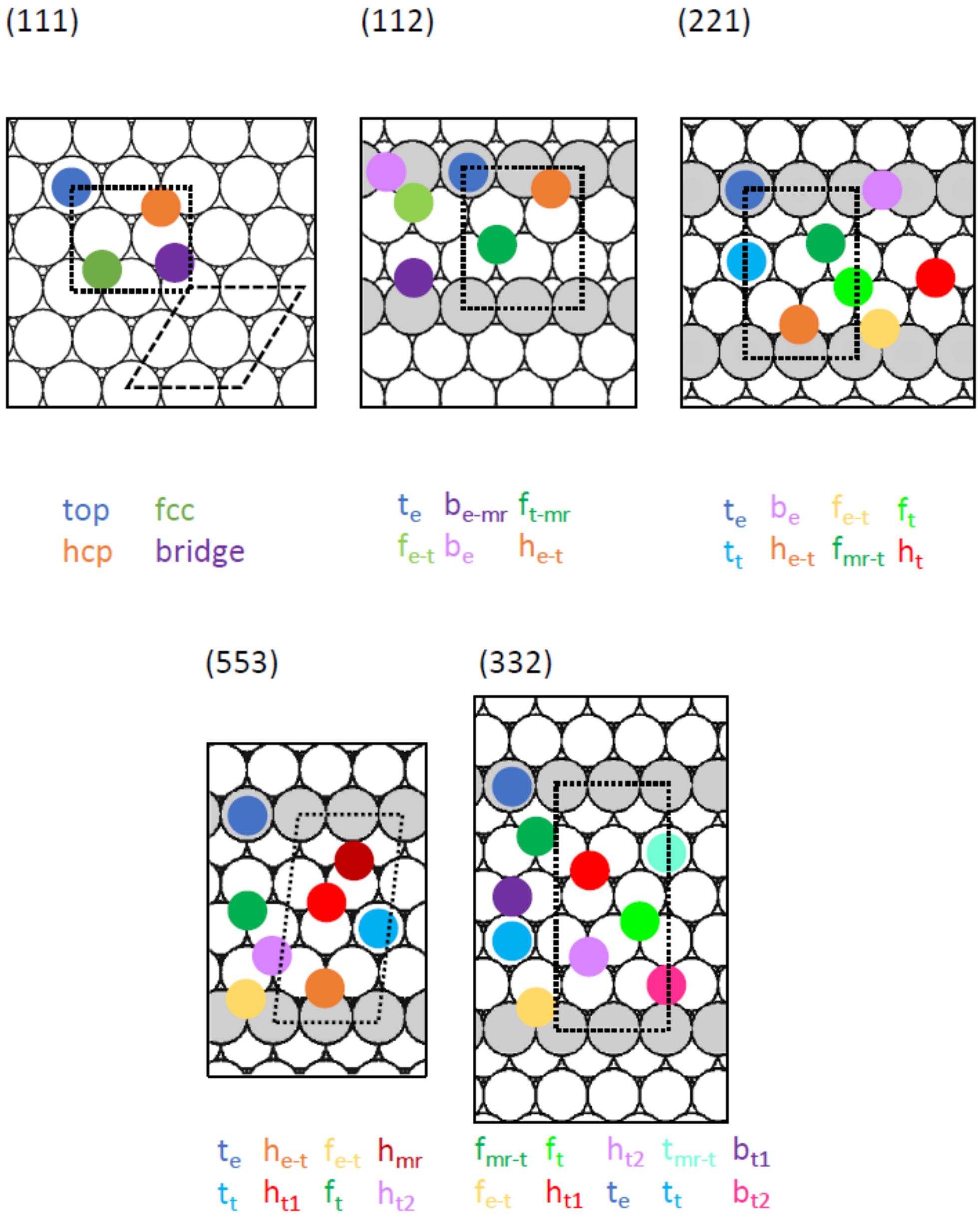

Figure S4: Considered adsorption sites on the (111), (112), (221), (553) and (332) surfaces. The step is indicated in grey. Unit cells are drawn with black dotted lines. Colors are indicated as below with t: top, h: hollow hcp, f: hollow fcc and b: bridge site. The subscript describes where the atom is placed to distinguish different kind of sites.

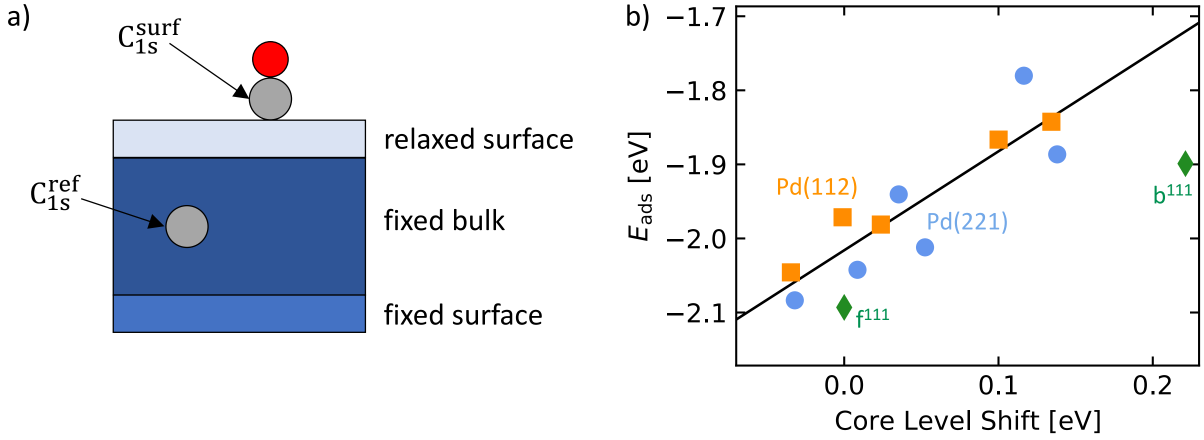

Figure S5: a) Scheme of the CLS C 1s calculation. b) CLS relative to C 1s at Pd(111) fcc ($\theta = 1/9$) for Pd(111): green diamonds, Pd(112): orange squares and Pd(221): blue circles. The line is a linear fit of the data.

References

(1) Schiller, F.; Ilyn, M.; Pérez-Dieste, V.; Escudero, C.; Huck-Iriart, C.; Ruiz del Arbol, N.; Hagman, B.; Merte, L. R.; Bertram, F.; Shipilin, M. et al. Catalytic Oxidation of Carbon Monoxide on a Curved Pd Crystal: Spatial Variation of Active and Poisoning Phases in Stationary Conditions. *Journal of the American Chemical Society* **2018**, *140*, 16245–16252.

(2) Ilyn, M.; Magaña, A.; Walter, A. L.; Lobo-Checa, J.; De Oteyza, D. G.; Schiller, F.; Ortega, J. E. Step-doubling at Vicinal Ni(111) Surfaces Investigated with a Curved Crystal. *Journal of Physical Chemistry C* **2017**, *121*, 3880–3886.

(3) Blomberg, S.; Zetterberg, J.; Zhou, J.; Merte, L. R.; Gustafson, J.; Shipilin, M.; Trinchero, A.; Miccio, L. A.; Magaña, A.; Ilyn, M. et al. Strain Dependent Light-off Temperature in Catalysis Revealed by Planar Laser-Induced Fluorescence. *ACS Catalysis* **2017**, *7*, 110–114.

(4) Garcia-Martinez, F.; Schiller, F.; Blomberg, S.; Shipilin, M.; Merte, L. R.; Gustafson, J.; Lundgren, E.; Ortega, J. E. CO chemisorption on vicinal Rh(111) surfaces studied with a curved crystal. *The Journal of Physical Chemistry C* **2020**, *124*, 9305–9313.

(5) Ertl, G.; Koch, J. Adsorption von CO auf einer Palladium(111)-Oberfläche. *Zeitschrift für Naturforschung A* **1970**, *25*, 1906–1912.

(6) lmfit package, https://lmfit.github.io/lmfit-py/.

(7) Doniach, S.; Sunjic, M. Many-electron singularity i n x-ray photoemission andx-ray line spectra from metals. *J. Phys. C: Solid State Phys.* **1970**, *3*, 285–291.

(8) Shirley, D. A. High-resolution x-ray photoemission spectrum of the valence bands of gold. *Physical Review B* **1972**, *5*, 4709–4714.

(9) Surnev, S.; Sock, M.; Ramsey, M.; Netzer, F.; Wiklund, M.; Borg, M.; Andersen, J. CO adsorption on Pd(111): a high-resolution core level photoemission and electron energy loss spectroscopy study. *Surface Science* **2000**, *470*, 171–185.

(10) Martin, N. M.; Van den Bossche, M.; Grönbeck, H.; Hakanoglu, C.; Zhang, F.; Li, T.; Gustafson, J.; Weaver, J. F.; Lundgren, E. CO Adsorption on Clean and Oxidized Pd(111). *The Journal of Physical Chemistry C* **2014**, *118*, 1118–1128.

(11) Kresse, G.; Hafner, J. Ab Initio Molecular Dynamics for Liquid Metals. *Physical Review B* **1993**, *47*, 558–561.

(12) Kresse, G.; Hafner, J. Ab Initio Molecular-Dynamics Simulation of the Liquid-Metal—Amorphous-Semiconductor Transition in Germanium. *Physical Review B* **1994**, *49*, 14251–14269.

(13) Kresse, G.; Furthmüller, J. Efficient Iterative Schemes for Ab Initio Total-Energy Calculations using a Plane-Wave Basis Set. *Physical Review B* **1996**, *54*, 11169–11186.

(14) Perdew, J. P.; Burke, K.; Ernzerhof, M. Generalized Gradient Approximation Made Simple. *Phys. Rev. Lett.* **1997**, *78*, 1396.

(15) Blöchl, P. E. Projector augmented-wave method. *Phys. Rev. B - Condens. Matter Mater. Phys.* **1994**, *50*, 17953–17979.

(16) Kresse, G.; Joubert, D. From Ultrasoft Pseudopotentials to the Projector Augmented-Wave Method. *Physical Review B* **1999**, *59*, 1758–1775.

(17) Monkhorst, H. J.; Pack, J. D. Special points for Brillouin-zone integrations. *Phys. Rev. B.* **1976**, *13*, 5188–5192.

(18) Grinberg, I.; Yourdshahyan, Y.; Rappe, A. M. CO on Pt(111) puzzle: A possible solution. *Journal of Chemical Physics* **2002**, *117*, 2264–2270.

(19) Olsen, R. A.; Philipsen, P. H.; Baerends, E. J. CO on Pt(111): A puzzle revisited. *Journal of Chemical Physics* **2003**, *119*, 4522–4528.

(20) Feibelman, P. J.; Hammer, B.; Norskov, J. K.; Wagner, F.; Scheffler, M.; Stump, R.; Watwe, R.; Dumesic, J. The CO/Pt(111) Puzzle. *Journal of Physical Chemistry B* **2001**, *105*, 4018–4025.

(21) Conrad, H.; Ertl, G.; Koch, J.; Latta, E. E. Adsorption of CO on Pd single crystal surfaces. *Surface Science* **1974**, *43*, 462–480.

(22) Granda-Marulanda, L. P.; Rendón-Calle, A.; Builes, S.; Illas, F.; Koper, M. T. M.; Calle-Vallejo, F. A Semiempirical Method to Detect and Correct DFT-Based Gas-Phase Errors and Its Application in Electrocatalysis. *ACS Catalysis* **2020**, *10*, 6900–6907.

(23) Pehlke, E.; Scheffler, M. *Phys. Rev. Lett.* **1993**, *71*, 2338.

(24) Johansson, B.; Mårtensson, N. *Phys. Rev. B* **1980**, *21*, 4427.

(25) Ganduglia-Pirovano, M.; Natoli, V.; Cohen, M.; Kudrnovský, J. Potential, core-level, and d band shifts at transition-metal surfaces. *Physical Review B - Condensed Matter and Materials Physics* **1996**, *54*, 8892–8898.

(26) Lizzit, S.; Zhang, Y.; Kostov, K. L.; Petaccia, L.; Baraldi, A.; Menzel, D.; Reuter, K. O-and H-induced surface core level shifts on Ru(0001): Prevalence of the additivityrule. *Journal of Physics Condensed Matter* **2009**, *21*.

(27) Grönbeck, H.; Klacar, S.; Martin, N. M.; Hellman, A.; Lundgren, E.; Andersen, J. N. Mechanism for reversed photoemission core-level shifts of oxidized Ag. *Physical Review B - Condensed Matter and Materials Physics* **2012**, *85*, 1–5.

(28) Alexey A. Tal Alvaro Posada-Borbón, H. J. G.; Abrikosov, I. A. Correlation between Ethylene Adsorption Energies and Core-Level Shifts for Pd Nanoclusters. *J. Phys. Chem. C* **2019**, *123*, 2544–2548.

(29) Walter, A. L.; Schiller, F.; Corso, M.; Merte, L. R.; Bertram, F.; Lobo-Checa, J.; Shipilin, M.; Gustafson, J.; Lundgren, E.; Brión-Ríos, A. X. et al. X-ray photoemission analysis of clean and carbon monoxide-chemisorbed platinum(111) stepped surfaces using a curved crystal. *Nature Communications* **2015**, *6*, 8903.
